# Development and nutritional evaluation of local ingredients‐based supplements to treat moderate acute malnutrition among children aged below five years: A descriptive study from rural Wolaita, Southern Ethiopia

**DOI:** 10.1002/fsn3.1927

**Published:** 2020-10-05

**Authors:** Debritu Nane, Anne Hatløy, Bernt Lindtjørn

**Affiliations:** ^1^ School of Public and Environmental Health Hawassa University Hawassa Ethiopia; ^2^ Centre for International Health University of Bergen Bergen Norway; ^3^ Fafo Institute for Labour and Social Research Oslo Norway

**Keywords:** development, Ethiopia, local ingredients‐based supplement, moderate acute malnutrition

## Abstract

In Ethiopia, moderate acute malnutrition (MAM) is a persistent public health problem. The current management approaches for MAM among children are counseling in food‐secure settings and food supplementation in chronically food‐insecure areas. The objective of this study was to develop a local ingredients‐based supplement (LIBS) for treating MAM among children. Collection of food ingredients (pumpkin seed, amaranth grain, flaxseed, peanut, and emmer wheat) was made. Sorting, soaking, drying, roasting, and milling of ingredients were done. Nutrient analysis was done using triplicate measurements of each nutrient. One‐way ANOVA was used to analyze differences in means with ± standard deviation of nutrient measurements among the supplements. The nutrient content of four developed LIBS ranged from 20.3 g to 22.5 g for protein, 29.3 g to 33.5 g for fat, 509.5 kcal to 570.0 for kcal, 6.0 g to 8.5 g for fiber, 2.8 g to 3.7 g for moisture, and 2.1 g to 4.3 g for ash. The mineral and antinutrient components ranged from 75.6 mg to 115.6 mg for calcium, 473.1 mg to 570.2 mg for potassium, 79.3 mg to 114.4 mg for sodium, 4.1 mg to 5.6 mg for zinc, 8.2 mg to 10.2 mg for iron, 442.6 mg to 470.4 mg for phosphorous, and 2.1 mg to 4.3 mg for phytate. The LIBS with the highest portion of pumpkin seed had significantly highest amounts of protein, fat, calories, iron, zinc, and potassium. The results found were within the recommended range of required nutrients for the treatment of children with MAM. Therefore, LIBS may be used for the management of children with MAM.

## Graphical abstract

Moderate acute malnutrition is a persistent problem in developing country. The objective of this study was to develop a local ingredients‐based supplement for treating moderate acute malnutrition among children. The results found were within the recommended range of required nutrients for the treatment of children with moderate acute malnutrition.

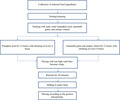

## 1 INTRODUCTION

Globally, moderate acute malnutrition (MAM) (weight for height < −2 to ≥ −3 Z scores) affects approximately 5% of children below 5 years of age (Black et al., 2013). MAM is widespread in developing countries and a persistent public health problem in Ethiopia (Adamu et al., 2017; Central Statistical Agency (CSA), 2017; ; ; ). The 2016 Ethiopian Demographic and Health Survey (EDHS) stated that 38 percent of children below five years of age were stunted, and 10 percent had acute malnutrition (CSA, 2017).

Children with MAM are more likely to suffer delays in their physical growth and cognitive development and are at greater risk of death than well‐nourished children (LaGrone et al., 2012). If children with MAM are not adequately managed, MAM can progress to severe acute malnutrition (SAM), which is a life‐threatening condition (James et al., 2016; World Health Organization (WHO), 2012; ; ; ; ; ; ; ; ; ).

Children with MAM have nutritional requirements that differ from nonmalnourished and SAM children (WHO, 2012). They need a high energy intake and essential nutrients to recover the existing deficiencies and to support normal growth (Amegovu et al., 2013; Karakochuk et al., 2012). Diets deficient in important nutrients, combined with a high burden of infectious disease, are among the underlying causes of malnutrition in young children (Nga et al., 2013). In 2012, the World Health Organization (WHO) stated that the provision of locally available, nutrient‐dense foods can help to improve the nutritional status of MAM children and prevent SAM (WHO, 2012).

A variety of food supplements are presently used to treat MAM (Medoua et al., 2015). These consist of corn‐soy blend (CSB) (de Pee & Bloem, 2009); BP5 biscuits (Medoua et al., 2015); and lipid‐based nutrient products (Ackatia et al., 2015). Of these therapeutic supplements, the most commonly used is fortified blended flour, mainly CSB prepared as porridge (Medoua et al., 2015; de Pee & Bloem, 2009). However, in addition to the high commodity cost, there are concerns about their long‐term sustainability (Isanaka et al., 2019; Nikie'ma et al., 2014).

In nutritional management of MAM, optimal feeding of locally accessible nutrient‐dense foods has been shown to be effective at the household level (Ashworth & Ferguson, 2009). These food supplements can be developed from locally available foods that are accessible to all the people and include the necessary amounts of nutrients. Local food ingredients are cheap and can provide key nutrients needed for the effective recovery of children with MAM (Andrew et al., 2013). Therefore, ready‐to‐use supplementary food (RUSF) made from locally available, nutritionally dense foods might have a positive effect on treating MAM children (Wagh & Deore, 2015).

In resource‐limited countries, counseling and either general or targeted distribution of CSB are often given as management of children with MAM (Nikie'ma et al., 2014; de Pee & Bloem, 2009). Unfortunately, the existing management strategy of MAM in Ethiopia is to limit supplementary feeding programs (SFPs) to selected districts of the country defined as chronically food‐insecure. There are no food supplementation programs for MAM children in food‐secure areas. In these districts, dietary counseling, vitamin A supplementation, and deworming are the strategies used to manage MAM children (James et al., 2016).

Local food materials can be cheap and provide essential nutrients required for the successful recovery of children with MAM. As far as our knowledge is concerned, there is no local development of supplementary food from local ingredients for the treatment of MAM in Ethiopia. On the other hand, WHO recommends the use of locally available, nutritionally dense foods for the treatment of children with MAM (WHO, 2012). In the current work, pumpkin seeds, peanuts, amaranth grain, flaxseeds, and emmer wheat were used in the formulation of four local ingredients‐based supplements (LIBS). This paper mainly focuses on the development and quality evaluation of these LIBS. The objective of this study was to develop a local ingredients‐based supplement for treating MAM among children.

## 2 METHODS

### 2.1 Selection of food ingredients

A checklist was prepared to list all food ingredients which are commonly consumed in the study area. Different international nutrient databases, such as the United States Department of Agriculture (USDA), the Food and Agriculture Organization (FAO), as well as national food databases (Ethiopian food composition table), were reviewed to determine the nutritive value of ingredients. The final selection of candidate food ingredients that have the potential to be developed into a supplement was done based on their availability, accessibility, cost, and nutritive value. All selected ingredients (pumpkin seeds, peanuts, amaranth grain, flaxseeds, and emmer wheat) were purchased from the local markets found in the Wolaita zone.

### 2.2 Supplement processing

**Figure 1:**
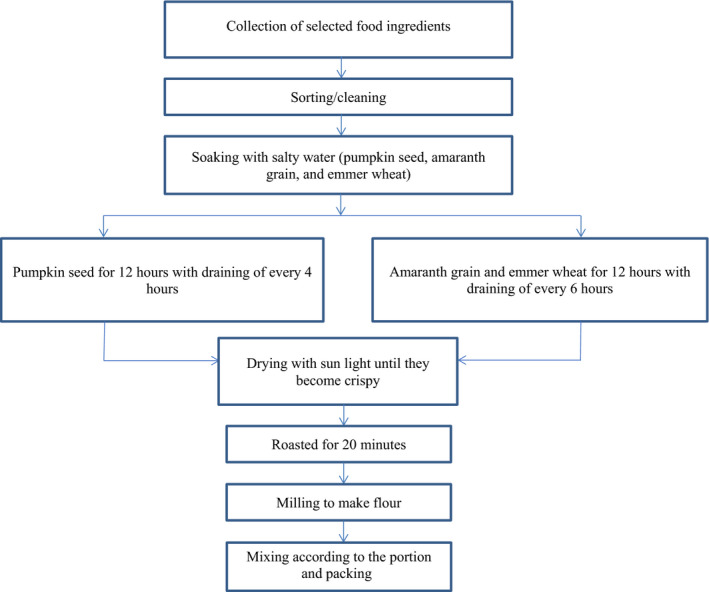
Processing flow of local ingredients‐based supplement. Collection of selected food ingredients = candidate food ingredients (pumpkin seed, amaranth grain, flaxseed, peanut, and emmer wheat) that have the potential to develop into supplement were collected. Sorting/cleaning = damaged seeds, foreign matter, and the shriveled and/or discolored seeds were removed from all food ingredients by hand‐sorting. Soaking in salty water (pumpkin seed, peanut, amaranth grain, and emmer wheat) = the cleaned pumpkin seed, peanut, amaranth grain, and emmer wheat were soaked in salty water. Pumpkin seed for 12 hr with draining every 4 hr; peanut for 6 hr = pumpkin seed was soaked in salty water for 12 hr with draining every 4 hr, and peanut was soaked for 6 hr. Amaranth grain and emmer wheat for 12 hr with the draining of every 6 hr = amaranth grain and emmer wheat were soaked in salty water for 12 hr with draining every 6 hr. Drying in sunlight until they become crispy = all ingredients (pumpkin seed, amaranth grain, flaxseed, peanut, emmer wheat) dried in sunlight until they become crispy. Roasted for 20 min = each dried ingredient was roasted at approximately 150°C for approximately 20 min. Milling to make flour = milling of individual ingredients was done using flour miller. Mixing according to the portion and packing = the flour of ingredients was mixed according to the determined portion of each ingredient to form the supplement and packed

All ingredients were hand‐sorted to remove damaged seeds, foreign matter, and shriveled and/or discolored seeds. The small‐sized debris was further removed by sieving with perforated shakers. The cleaned pumpkin seed, amaranth grain, and emmer wheat were washed thoroughly with tap water and soaked in salty water. Pumpkin seed was soaked for 12 hr, with draining every four hours. Peanut was soaked for 6 hr. Amaranth grain and emmer wheat were soaked for 12 hr, with draining every six hours. After draining the soaked ingredients, we spread them in the sunlight to dry until they become crispy. The peanut and flaxseed were wiped. Each ingredient was roasted separately at approximately 150°C for approximately 20 min, with continuous stirring, until it developed an aroma. The roasted ingredients were cooled. After cooling, the peanuts were skinned and separated from seeds by winnowing. Finally, the milling of individual ingredients was done using a flour miller (Figure 1).

### 2.3 Supplement development

Determinations of proportion of the supplements’ ingredients were done by Nutrisurvey computer software, employing linear programming. Four supplements (LIBS 1, LIBS 2, LIBS 3, and LIBS 4) were formed from the mixture, with different proportions of each ingredient (Table 1). The nutrient content of each formulation was dependent on composition, intended to match the recommended daily allowances for children aged 6 to 59 months (Golden, 2009; WHO, 2012).

**Table 1:** Proportion of ingredients within food samples/100 gm

Food samples (g)	Pumpkin seed (g)	Peanut (g)	Amaranth (g)	Flaxseed (g)	Emmer wheat (g)
LIBS 1	20	20	20	20	20
LIBS 2	25	20	15	20	20
LIBS 3	25	15	20	25	15
LIBS 4	30	25	20	15	10

Abbreviations: g, gram; LIBS, local ingredients‐based supplement.

### 2.4 Nutrient analysis

The nutrient analysis was done at the Ethiopian Health and Nutrition Research Institute (EHNRI) and Hawassa University Food Science Laboratory.

#### 2.4.1 Proximate analysis

The standardized procedure of the Association of Official Analytical Chemists (AOAC) was followed to estimate the nutrient composition of the supplements (AOAC, 2005). Triplicate measurement of nutrients was maintained. The moisture content of supplements is significant for their shelf life, with better storage stability maintained by the lower moisture content of the supplement (Sharma & Lakhawat, 2017). The moisture content of the LIBS was determined using AOAC Official Method 925.10 (McCleary et al., 2013), while the determination of ash content was made using AOAC Official Method 923.03—Direct method (AOAC, 2000). The crude fiber content was determined by AOAC Official Method 962.09—Ceramic Fiber Filter method (Derib et al., 2018). The crude fat content was analyzed by using AOAC Official Method 920.39—Soxhelt (AOAC, 2000). Determination of crude protein content was made by ESISO 1871:2013 test method (Derib et al., 2018). Carbohydrate content was calculated by difference including fiber: CHO% = 100‐(moisture content% + crude protein% + fat% + fiber% + ash %). Determination of energy was done by using the Atwater's conversion factor: 4 kcal/g for carbohydrate, 4 kcal/g for protein, and 9 kcal/g for fat (Food and Agriculture Organization (FAO), 2003).

#### 2.4.2 Mineral and antinutrient analysis

Calcium content was determined by using the AOAC Official Method 923.03—EDTA titration (AOAC, 2000; Wu & Wu, 2017). Iron, zinc, potassium, and phosphorus contents were determined by using atomic absorption/emission spectrophotometer equipment. Phytate was determined by using the Latta and Eskin method (Latta & Eskin, 1980), as modified by Vaintraub and Lapteva (1988).

#### 2.4.3 Determination of the molar ratio of phytate to mineral

In this study, phytate‐to‐mineral molar ratios were used to determine the inhibitory effects of phytate on the bioavailability of minerals. The molar ratio of phytate and minerals was determined by dividing the weight of phytate and minerals with its atomic weight. Phytate‐to‐mineral mole ratios were calculated as follows (Borquaye et al., 2017; Gargari et al., 2007):

$$\text{Molar}\;\text{ration}=\frac{\text{Moles}\;\text{of}\;\text{anti}‐\text{nutrient}}{\text{Moles}\;\text{of}\;\text{mineral}}$$

The molar mass of the phytate used was 660 g/mol. The recommended critical values used in this work are (phytate: Zn)> 15, (phytate: Fe)> 1, and (phytate: Ca) >0.24 (Borquaye et al., 2017).

### 2.5 Quality control

The food ingredients, as well as developed supplements, were stored in areas that were clean and free of rodents. Supplement processing workers received food hygiene training before starting the supplement development process. Constant supervision of supplement processors was done. They received regular medical checkups throughout the supplement development. They also washed and thoroughly dried their hands before processing the supplement and wore clean plastic gloves, hair coverings, and protective coats during supplement development. To prevent the introduction of water into LIBS during development, we restricted the frequency with which the processing equipment was cleaned with soap and water and simply dry‐wiped it clean as an alternative.

### 2.6 Statistical analysis

Data analysis was carried out using the Statistical Package for the Social Sciences (SPSS 20). Values were expressed as grams with means of triplicates ± standard deviation. The comparison of the difference in proximate, mineral, and antinutrient content among food groups was analyzed using a one‐way analysis of variance (ANOVA) and post hoc Tukey's test. The significance of differences was considered at 0.05 level of probability (*p* < .05).

## 3 RESULTS AND DISCUSSION

### 3.1 Proximate composition

#### 3.1.1 Protein content

The protein content of the four food supplements ranged from 20.3 g to 22.6 g. LIBS 4 (22.6 g) had significantly higher protein content than the others (Table 2). The protein content of all the four LIBS is within the recommended values of protein for the management of MAM (WHO, 2012). The protein content of LIBS 4 was possibly a result of using the highest proportions of pumpkin seed and peanut while developing this supplement. Pumpkin seed and peanut are good sources of high‐quality protein and might contribute considerably to the recommended human daily protein allowance (Settaluri et al., 2012; Sharma & Lakhawat, 2017). Since these two ingredients have a higher protein content, it might be assumed that the addition of pumpkin seed and peanut in this therapeutic supplement has the potential to overcome protein‐energy malnutrition.

**Table 2:** Proximate composition of food supplements/100 gm

Components	LIBS 1	LIBS 2	LIBS 3	LIBS 4
Protein (g)	20.7 ± 0.2^b^	21.3 ± 0.3^b^	20.3 ± 0.6^b^	22.6 ± 0.3^a^
Fat (g)	29.3 ± 0.4^c^	31.3 ± 4^b^	31.6 ± 0.7^b^	33.5 ± 0.04^a^
CHO (g)	40.0 ± 0.4^a^	37.3 ± 0.2^b^	37.5 ± 0.5^b^	35.4 ± 1.4^c^
Fiber (g)	6.5 ± 0.2^b^	8.3 ± 0.3^a^	6.0 ± 0.6^b^	6.0 ± 0.2^b^
Moisture (g)	3.6 ± 0.1^a^	3.73 ± 0.2^a^	2.8 ± 0.3^b^	3.0 ± 0.1^b^
Ash (g)	2.47 ± 0.1^c^	3.6 ± 0.3^b^	4.3 ± 0.1^a^	2.1 ± 0.03^d^

Abbreviations: CHO, carbohydrate; LIBS, local ingredients‐based supplement; g, gram.

Values are means of triplicates ± standard deviation. Values with a different superscript in a row are significantly different (*p* < .05).

#### 3.1.2 Fat content

The fat content of the four food supplements ranged from 29.3 g to 33.5 g. The least amount was found in LIBS 1 (29.3 g), whereas the highest amount of fat was found in LIBS 4 (33.5 g). There was no significant difference in fat content between LIBS 2 and LIBS 3. LIBS 1 was significantly lower in fat content than the other LIBS, whereas LIBS 4 was significantly higher than the others (Table 2). The fat content of all the LIBS is within the range of recommended fat content of therapeutic supplements for MAM (WHO, 2012). LIBS 4 having the highest amount of fat might be due to using the highest proportion of pumpkin seed while formulating it. Pumpkin seed is quite rich in crude fat and oil (Sharma & Lakhawat, 2017). Children with MAM have high energy requirements needing a diet with adequate fat content, which is also required for the absorption of vitamins A and E (Michaelsen et al., 2009). Therapeutic supplements must contain an adequate amount of fat to deliver the required energy to the malnourished child.

#### 3.1.3 CHO and energy content

In this study, the CHO content of the supplements ranged from 35.4.0 g to 40.0 g. The mean values for LIBS 2 and LIBS 3 were similar. The CHO content of LIBS 1 was significantly higher than the other LIBS (Table 2). The energy values ranged from 509.5 kcal to 532.0 kcal. The energy level of LIBS 4 was significantly higher than the other supplements, whereas LIBS 1 was the lowest. There were no significant differences between LIBS 1, LIBS 2, and LIBS 3. The high energy value in LIBS 4 was possibly as a result of this supplement having the highest amount of fat and protein in it. Concentrated energy is an essential quality of foods developed for children with MAM, given their increased energy requirements (Singh et al., 2011). The energy contents of all four LIBS were beyond the indicated minimum amount of 380 kcal for fortified blended foods (Amegovu et al., 2013).

#### 3.1.4 Fiber content

The fiber content of the LIBS ranged from 6.0 g to 8.3 g (Table 2). LIBS 2 (8.3 g) had significantly higher fiber content than all other supplements. The high fiber content for LIBS 2 might be due to it having the highest proportion of flaxseed. Flaxseed contains a very high amount of dietary fiber, making it important in the human diet (Singh et al., 2011). Dietary fiber plays a vital role in the digestion process. Mainly, soluble fiber conveys prebiotic properties, whereas insoluble fiber averts constipation (Amegovu et al., 2013). However, constipation is not the most important concern in malnourished children. This leads to recommending a low intake of insoluble fiber in the diets but a high intake of soluble fiber in children. Unfortunately, no limits have been set due to limited evidence on problems caused by insoluble fiber in children (Michaelsen et al., 2009).

#### 3.1.5 Moisture content

The moisture content of the four LIBS ranged from 2.8 g to 3.7 g. There was no significant difference in the moisture content of LIBS 1 and LIBS 2. Also, there was no significant difference in the moisture content of LIBS 3 and LIBS 4. However, LIBS 1 and LIBS 2 were significantly different from LIBS 3 and LIBS 4 (Table 2). This might be because the moisture content depends on the hydroscopic capacity of the seed, and we used the equal/nearly equal portions of individual ingredients for making LIBS 1 and LIBS 2, and for LIBS 3 and LIBS 4.

The highest moisture content was found in LIBS 2, and the lowest was found in LIBS 3 (Table 2). The moisture content is within the recommended level for the proper storage of dehydrated foodstuff (Codex Alimentarius Commission, 2006).

#### 3.1.6 Ash content

Total ash content ranged from 2.1 g to 4.3 g. It was significantly different for all supplements. The lowest ash content was found in LIBS 4 (Table 2).

### 3.2 Mineral and antinutrient composition

#### 3.2.1 Calcium content

The calcium content was significantly different among the four supplements. It ranged from 104.6 mg to 115.6 mg/100 g. The calcium content was highest in LIBS 3 (Table 3). The values for all the LIBS are lower than the recommended calcium levels for children with MAM (Amegovu et al., 2014).

**Table 3:** Mineral and antinutrient composition of food samples/100gm

Parameters	LIBS 1	LIBS 2	LIBS 3	LIBS 4
Calcium (mg)	104.6 ± 0.05^c^	111.1 ± 0.2^b^	115.6 ± 0.15^a^	107.0 ± 2.0^c^
Potassium (mg)	553.4 ± 0.4^d^	568.0 ± 0.5^c^	601 ± 0.3^b^	666.1 ± 0.2^a^
Phosphorous (mg)	442.6 ± 0.8^c^	444.6 ± 2.3^c^	454.5 ± 2.2^b^	470.4 ± 0.7^a^
Iron (mg)	8.2 ± 0.1^c^	9.0 ± 0.2^b^	8.9 ± 0.5^b^	10.2 ± 0.1^a^
Sodium (mg)	104.4 ± 0.03^a^	98.01 ± 0.08^c^	79.4 ± 0.1^d^	88.3 ± 0.3^b^
Zinc (mg)	4.1 ± 0.1^b^	4.5 ± 0.2^b^	4.9 ± 0.01^b^	5.6 ± 0.2^a^
Magnesium (mg)	176.2 ± 0.2^d^	206 ± 0.2^a^	189.4 ± 0.1^c^	200.5 ± 0.5^b^
Phytate (mg)	2.47 ± 0.1^c^	3.6 ± 0.3^b^	4.3 ± 0.1^a^	2.1 ± 0.03^d^

Abbreviations: LIBS, local ingredients‐based supplement; mg, milligrams.

Values are means of triplicates ± standard deviation. Values with a different superscript in a row are significantly different (*p* < .05).

#### 3.2.2 Potassium content

The potassium content was significantly different among the supplements. It ranged from 553.4 mg to 666.1 mg/100 g. The highest potassium content was found in LIBS 4 (666.1 mg/532 kcal) (Table 3). These levels were relatively similar to the recommended value (1,400 mg/1,000 kcal) (Golden, 2009). All malnourished children have depleted potassium levels. Supplementary foods should contain an adequate amount of potassium to maintain renal and fecal excretion of it (Amegovu et al., 2013).

#### 3.2.3 Phosphorus content

The phosphorous values ranged from 442.6 mg to 470.4 mg/100 g (Table 3). The least amount of phosphorus was found in LIBS 1, whereas the highest content was found in LIBS 4. These values were similar to the values of phosphorus indicated in the study done by Amegovu and colleagues (Amegovu et al., 2014).

#### 3.2.4 Iron content

The iron content was significantly different among supplements. It ranged from 8.2 mg to 10.2 mg/100 g. The highest iron content was found in LIBS 4 (10.2 mg/532 kcal), whereas the lowest was found in LIBS 1 (8.2 mg/510 kcal) (Table 3). These values were higher than the recommended levels of iron for the management of MAM (9 mg/1,000 kcal) by Golden (2009). This might be because the ingredients that we used for the development of LIBS 4 were rich in iron.

#### 3.2.5 Sodium content

The sodium content of the four LIBS ranged from 79.4 mg to 104.4 mg/100 g. The highest amount of sodium was found in LIBS 1 (104.4/510 kcal), whereas the lowest sodium content was found in LIBS 3 (79.4 mg/515 kcal) (Table 3). During malnutrition, the total body sodium is considerably increased. Foods that contain high sodium levels would then be nonbeneficial (Amegovu et al., 2013). In this study, the values for sodium did not exceed the maximum recommended level (500 mg/1,000 kcal) set by WHO (2012).

#### 3.2.6 Zinc content

The zinc content for the four supplements ranged from 4.1 mg to 5.6 mg/100 g. The zinc content in LIBS 4 was significantly highest, whereas the lowest amount was found in LIBS 1. There were no statistical differences in the zinc contents of LIBS 1, 2, and 3 (Table 3). Zinc is an important mineral that helps in preventing diarrhea in malnourished children (Michaelsen et al., 2009). In this study, the zinc values of the supplements were higher than the food blends used for the management of MAM and close to the recommended values for zinc (Amegovu et al., 2013; Golden, 2009).

#### 3.2.7 Magnesium content

In this study, the magnesium content of the four supplements varied significantly. It ranged from 176.2 mg to 206 mg/100 g. The lowest amount was found in LIBS 1, whereas highest amount in LIBS 2 (Table 3). The magnesium content found in these newly developed supplements was in line with the study done by Stobaugh HC (Stobaugh et al., 2016), and comparable with the recommended values of magnesium for children with MAM ( Golden, 2009; WHO, 2012).

#### 3.2.8 Phytate content

The phytate levels of all supplements were significantly different (Table 3). The least amount of phytate was found in LIBS 4.

#### 3.2.9 Phytate‐to‐mineral ratios

Phytate‐to‐zinc, phytate‐to‐iron, and phytate‐to‐calcium molar ratios were below the recommended critical values for any supplement (Table 4). Phytate is known to reduce the bioavailability of minerals, mainly Zn, Ca, Mg, and Fe because it has a strong binding affinity to minerals. When a mineral binds to phytic acid, it converts into insoluble form, precipitates, and will not be absorbable in the intestines. Thus, its high intake can cause mineral deficiency and undesirable (Gargari et al., 2007; Hurrell, 2003). The normal level of phytate‐to‐mineral molar ratio identified in this study showed that there is a good bioavailability of minerals for all newly formulated supplements. This might be because of soaking the ingredients with salty water for several hours as a supplement processing step. As a study done by Ochola, food processing steps like soaking of grains and germinating can reduce the phytic acid (Ochola, 2013).

**Table 4:** Comparison of antinutrient‐to‐mineral molar ratio with recommended critical values

Molar ratio	LIBS 1	LIBS 2	LIBS 3	LIBS 4	Critical values
Phytate:Zn	0.06^c^	0.08^b^	0.09^a^	0.04^d^	15
Phytate:Fe	0.03^b^	0.03^b^	0.04^a^	0.02^c^	1
Phytate:Ca	0.002^a^	0.002^a^	0.002^a^	0.001^b^	0.24

Note

Abbreviations: LIBS, local ingredients‐based supplement.

Values with a different superscript in a row are significantly different (*p* < .05).

#### 3.2.10 Comparison of the nutrient composition of LIBS 4 with the recommended levels for children with MAM

Among the newly formulated supplements, LIBS 4 is differently high in protein, fat, energy, iron, zinc, phosphorous, and potassium. The other nutrients of LIBS 4 such as magnesium, calcium, and sodium had lower values compared with the highest values among LIBSs. The amount of these nutrients were comparable with the recommended daily allowances for children aged 6–59 months with MAM, and it is also comparable with the conventional therapeutic food (CSB+) for the treatment of children with MAM (Amegovu et al., 2013; Golden, 2009; WHO, 2012) (Table 5).

**Table 5:** Nutrient composition of the supplementary foods with the established recommendations

Nutrients	LIBS 4 per 1,000 kcal	CSB + per 1,000 kcal	Recommended values per 1,000 kcal
Protein (gm)	42.5	40	20–43
Fat (gm)	63	25.4	25–65
Iron (mg)	19	39	13–20
Zn (mg)	10.5	9.9	20
Calcium (mg)	201	2,117	1,000–1,400
Phosphorous (mg)	884	1,152	850–1,400
Potassium (mg)	1,252	1,487	1,600
Magnesium (mg)	377	170.5	300
Sodium	166	65	<500

Abbreviations: CSB+, corn‐soy blend plus; mg, milligram; LIBS, local ingredients‐based supplement.

## 4 CONCLUSIONS AND RECOMMENDATIONS

Supplementary food for children with MAM aged 6–59 months, as evaluated in the present study, has been successfully developed using pumpkin seed, peanut, amaranth grain, flaxseed, and emmer wheat, which are locally available ingredients. The results found were within the recommended range of required nutrients except for calcium. Specifically, LIBS 4 contains an optimal amount of nutrients desirable for the treatment of children with MAM. LIBS 4 may, therefore, be used for the management of children with MAM in the rural community.

There is a need to determine the shelf life of the developed supplements and do a cost analysis of the superior supplement. Clinical trials need to be carried out to test the effectiveness of the developed supplements compared with other supplementary food in treating MAM among children. Fortification of the formulation with micronutrients such as ascorbic acid and calcium is also recommended. Production of ingredients should be enhanced to make ingredients readily available and ensure sustainability.

## CONFLICT OF INTEREST

All authors declare that they have no competing interests.

## ETHICAL REVIEW

This study was approved by the Hawassa University College of Medicine and Health Sciences Institutional Review Board (IRB/024/10) and regional committees for medical and health research ethics in Norway (2018/69/REK vest).
